# MIIST305 mitigates gastrointestinal acute radiation syndrome injury and ameliorates radiation-induced gut microbiome dysbiosis

**DOI:** 10.1080/19490976.2025.2458189

**Published:** 2025-02-10

**Authors:** Debmalya Mitra, Gabriel K. Armijo, Elizabeth H. Ober, Shenda M. Baker, Helen C. Turner, Constantinos G. Broustas

**Affiliations:** a Center for Radiological Research, Columbia University Vagelos College of Physicians and Surgeons, Columbia University Irving Medical Center, New York, NY, USA; b Synedgen Inc., Claremont, CA, USA

**Keywords:** Gastrointestinal-acute radiation syndrome, radiomitigator, MIIST305, gut microbiota, inflammation

## Abstract

High-dose radiation exposure results in gastrointestinal (GI) acute radiation syndrome identified by the destruction of mucosal layer, intestinal growth barrier dysfunction, and aberrant inflammatory responses. Further, radiation causes gut microbiome dysbiosis characterized by diminished microbial diversity, mostly commensal bacteria, and the spread of bacterial pathogens that trigger the recruitment of immune cells and the production of pro-inflammatory factors that lead to further GI tissue damage. Currently, there are no U.S. Food and Drug Administration (FDA) approved countermeasures that can treat radiation-induced GI injuries. To meet this critical need, Synedgen Inc. has developed a glycopolymer radiomitigator (MIIST305) that is specifically targeted to the GI tract, which acts by intercalating into the mucus layer and the glycocalyx of intestinal epithelial cells that could potentially ameliorate the deleterious effects of radiation. Male C57BL/6J adult mice were exposed to 13 Gy partial body X–irradiation with 5% bone marrow shielding and MIIST305 was administered on days 1, 3, and 5 post-irradiation. Approximately 85% of the animals survived the irradiation exposure and were apparently healthy until the end of the 30-day study period. In contrast, no control, Vehicle-treated animals survived past day 10 at this radiation dose. We show that MIIST305 improved intestinal epithelial barrier function and suppressed systemic inflammatory responses mediated by radiation-induced pro-inflammatory cytokines. Taxonomic profiling and community structure of the fecal and colonic mucosa microbiota demonstrated that MIIST305 treatment increased microbial diversity and restored abundance of beneficial commensal bacteria, including *Lactobacillus* and *Bifidobacterium* genera while suppressing potentially pathogenic bacteria *Enterococcus* and *Staphylococcus* compared with Vehicle-treated animals. In summary, MIIST305 is a novel GI-targeted therapeutic that greatly enhances survival in mice exposed to lethal radiation and protects the GI tract from injury by restoring a balanced gut microbiota and reducing pro-inflammatory responses. Further development of this drug as an FDA-approved medical countermeasure is of critical importance.

## Introduction

Accidental or intentional exposure to high-dose radiation often leads to severe hemorrhage, multiorgan failure, infection, sepsis, and death.^1^ The hematopoietic system and the gastrointestinal (GI) tract are particularly vulnerable to radiation-induced damage.^2,3^ High-dose radiation exposure can trigger a GI subsyndrome characterized by disruption of the mucosal layer, intestinal epithelial barrier dysfunction, and aberrant inflammatory responses potentially leading to rapid death.^3^ While advancements have been made to counteract the immediate effects of hematopoietic acute radiation syndrome (ARS),^1^ no FDA-approved medical countermeasures (MCMs) currently exist that can treat radiation-induced GI injuries.^4^

The lower GI tract harbors a rich community of microorganisms that reside within the luminal and mucosal compartments, which represent distinct niches differing in microbial diversity and composition and play crucial roles in intestinal physiology.^5^ A diverse and healthy community of commensal bacteria regulates key epithelial cell functions, contributing to the maintenance of intestinal epithelial barrier integrity and modulating the host’s inflammatory state under both homeostatic conditions and in response to injury.^6^ In contrast, microbiome dysbiosis promotes aberrant immune responses and susceptibility to inflammatory diseases.^7^ The diversity and composition of bacterial flora in the small and large intestine undergoes significant alterations in response to a single, high-dose, irradiation and, conversely, luminal microbiota composition influences the host’s intestinal response to radiation^8–11^ and contributes to increased sensitivity of the gut to inflammation.^12^ In rodent studies of high-dose radiation exposure (5–18 Gy), specific alterations in the microbiota have been observed, which include increased abundance of the phylum Proteobacteria, family Lactobacillaceae, Muribaculaceae and Prevotellaceae alongside decreased abundance of families Lachnospiraceae, Ruminococcaceae, and Clostridiaceae^13–18^

The intestinal epithelial barrier is protected from commensal and pathologic bacteria by three defense mechanisms: the mucus layer,^19,20^ the glycocalyx, a 3D matrix rich in carbohydrates and transmembrane mucins on the apical surface of intestinal epithelial cells (IECs),^21^ and the tight junctions^22^ between intestinal epithelial cells. The mucus layer acts as a first line of defense by preventing luminal bacteria from interacting with the intestinal barrier, thus reducing bacterial exposure of epithelial and immune cells.^23^ The primary component of the intestinal mucus is gelforming, O-linked glycosylated Muc2 polymers, which are predominantly secreted by goblet cells, and the gut microbiome has the capacity to alter the production of mucus.^24^ These Muc2 polymers are essential for maintaining a stable microbial community in the gut^25^ and support the expression of tight junctional proteins.^26^ Goblet cell depletion leads to mucus layer impairment, infection, inflammation, and aberrant cytokine response in the gut, further leading to improper expression of tight junction proteins that appear to be a component of several GI diseases.^27^ Even if a pathogenic microorganism manages to penetrate the mucus layer and reach the IEC, the surface glycocalyx will physically inhibit contact between microbes and IECs.^28,29^

With the hypothesis that the glycocalyx is a key therapeutic target for GI-ARS and to meet the critical need for GI-specific MCMs, Synedgen Inc. (Claremont, CA), has developed an orally delivered glycopolymer radiomitigator (MIIST305) that is specifically targeted to the GI tract to potentially alleviate the deleterious effects of radiation. MIIST305 is a polycationic glycopolymer from Synedgen’s multivalent innate immune signaling target platform (MIIST). As a GI-surface targeted therapeutic, MIIST305 associates with the mucus and epithelial glycocalyx.^30,31^ These anionic glycopolymers physically impede a vast majority of microorganisms from accessing the epithelial barrier,^28,29^ thus maintaining tolerance toward the commensal gut microbiota. The drug is administered orally and is not absorbed systematically, thus limiting the potential for possible systemic side-effects. Further, extensive non-clinical toxicology and safety pharmacology studies in two animal models have shown no adverse events at maximum deliverable doses of the drug.

In this study, we examined the efficacy of MIIST305 to alleviate GI-ARS pathophysiology in male C57BL/6 J mice. We assessed animal survival up to 30 d, following a single high-dose radiation exposure and showed that MIIST305 significantly reduced mortality. To simulate a realistic radiation exposure scenario, mice were irradiated with 5% bone marrow shielding (BM5), which approximates inhomogeneous radiation exposure likely to occur in a real-world nuclear event, where random shielding would be provided by structures such as buildings.^32,33^ Furthermore, we investigated histological injury, intestinal barrier function, and systemic and local cytokine production after radiation exposure. Finally, we analyzed the impact of radiation on the gut luminal and mucosal microbiome diversity and composition in MIIST305- or Vehicle-treated mice.

## Materials and methods

### Ethics statement

The animal studies were reviewed and approved by the Columbia University Irving Medical Center-Institutional Animal Care and Use Committee (CUIMC-IACUC), and all experimental procedures were conducted in accordance with CUIMC-IACUC guidelines and regulations (Protocol no. AABU0652).

### Animals

Eight-week-old male C57BL/6 J mice were obtained from Jackson Laboratory (Bar Harbor, ME,) and housed in the animal facilities at CUIMC under specific pathogen-free conditions and five animals were kept per individually ventilated cages. Mice were allowed to acclimate for 10 d prior to experimentation. The animal rooms were maintained at a temperature of 20 ± 3°C and a humidity of 40–60%, with a 12-h light/dark cycle. The mice were provided with 5053 Irradiated PicoLab® Rodent Diet 20 (Lab Diet®, Arden Hills, MN) and water ad libitum. Wet food pellets and hydrogel packs (Clear H_2_O, Westbrook, ME) were introduced to the cage floor following irradiation. Animals were randomly assigned into four treatment groups: (1) Vehicle unirradiated (UI), (2) MIIST305 UI, (3) Vehicle irradiated (IR), and (4) MIIST305 IR. Animals were identified by ear punches and cage labels.

### Irradiation

The animals from Vehicle IR and MIIST305 IR groups were irradiated with X-rays using an XRAD320 irradiator (Precision, Madison, CT), operated at 320 kV,12.5 mA. The table height was set at 52 cm, and the X-rays were administered at doses of 13.0 Gy and 12.5 Gy, with a dose rate of approximately 2.0 Gy/min as detailed in Table 1. Dosimetry was performed using a Radcal ion chamber (Monrovia, CA) to monitor the dose rate. Mice were sedated with isoflurane (Covetrus, Portland, ME) and placed in holding fixtures with the left hind leg extended out and shielded with a 0.6 cm thick lead block covering the femur, tibia, fibula, and paw, resulting in BM5.^34,35^ All irradiations were completed between 10:00 am–12:00 pm to avoid confounding circadian conditions.^36^ The mice from Vehicle UI and MIIST305 UI were sham irradiated.

**Table 1.** Comprehensive overview of irradiation procedure employed in the experimental study.

**Irradiation**	
Instrument	X-RAD320 (320 kV, 12.5 mA)
Radiation dose	13.0 Gy
Radiation dose rate	2.0 ± 0.1 Gy/min
Filter	1 (2 mm Al)
Dose rate calibration	RadCal ionization chamber (10X6-6) connected to an Accu-Dose+ digitizer module.
SSD	52 cm
PBI	5%
Mice (male C57BL/6 J, 8–10 wks)	Anesthetized by isoflurane
**MIIST305 treatment**	
Concentration/mouse	50 mg/kg delivered in 100 µL water
Control mice	100 µL sterile purified water
Delivery route	Oral gavage
Dosing	Days 1, 3, 5 post-irradiations

### Administration of MIIST305

Mice from the MIIST305 (UI/IR) groups were administered MIIST305 (50 mg/kg/day) diluted in sterile endotoxin-free purified water (Sigma-Aldrich, St. Louis, MO) via oral gavage, starting 24 h post irradiation with two further treatments on days 3 and 5. The Vehicle groups received only purified water. The volume of the dosage administered to each mouse was 100 µl.

### Health monitoring

The body weights of the animals were recorded prior to radiation exposure, after which they were returned to their respective cages and monitored once or twice daily until 14 d post irradiation (DPI) and once every other day thereafter for signs of body weight loss. Animals that lost 35% of their body weight or displayed signs of morbidity according to the Mouse Interventional Scoring System 3^37^ were euthanized via CO_2_ inhalation followed by cervical dislocation, in accordance with CUIMC-IACUC guidelines.

### Collection of blood and tissue sample

After euthanasia, blood samples were collected by cardiac puncture along with luminal and mucosal samples at 6 and 12 DPI timepoints for further analysis. A minimum of five mice/treatment group were considered for each timepoint.

### Histopathology

The colon was dissected from the small intestine at the ileocecal junction, washed in ice-cold phosphate buffered saline (PBS), and the length was measured in centimeters for each experimental group.^38^ Colon sections were prepared as Swiss rolls and fixed in a 10% neutralbuffered formalin solution for 24 h, followed by incubation with 70% ethanol. Tissue samples were submitted to the CUIMC Molecular Pathology Core Facility for sectioning and staining for crypt cell proliferation (Ki67) and goblet cell loss.^39^

### Immunohistochemistry (Ki67)

Colon tissue samples embedded in paraffin^40^ were dewaxed using xylene and subsequently hydrated. Antigen retrieval was performed, and endogenous peroxidase activity was inhibited with 3% H_2_O_2_. Slides were blocked with 5% BSA blocking buffer at room temperature for 25 min. Subsequently, tissues were incubated with Ki67 polyclonal (Cell Signaling Technology, Inc, Danvers, MA) antibody cat no. 12202S at 37°C for 2 h followed by anti-rabbit IgG incubation at 37°C for 1.5 h. Immunostaining was visualized using DAB/peroxidase substrate solution (Dako, Agilent, Santa Clara, CA), and samples were counterstained with hematoxylin at room temperature for 10–30 seconds.^41^ The slides were examined using a light microscope (10X objective lens; Echo Revolve, San Diego, CA), and the average number of crypts per field and the number of Ki67-positive cells per crypt was counted across five fields per slide for comparative analysis among the test groups.

### Mucin staining by Alcian blue

Dewaxed and rehydrated colonic tissue samples were stained using the Alcian blue Stain Kit (Vector Laboratories, Newark, CA) according to the manufacturer’s protocol and examined microscopically using a 10X objective.^42^ The intensity of Alcian blue staining was quantified by assessing the mean gray scale using ImageJ 1.53 (NIH, Bethesda, MD). Additionally, the average number of goblet cells was counted across five fields per slide for comparative analysis among the experimental groups.^43^

### Intestinal permeability assay

Test animals were fasted for 4 h, followed by oral gavage administration of FITC-dextran 4 kDa (Sigma-Aldrich) at a dose of 44 mg/100 g body weight to the experimental mice from the Vehicle IR and MIIST305 IR groups. Mice from the Vehicle UI group received PBS. After 4 h, mice were euthanized, and whole blood was collected by cardiac puncture. The FITC concentration in the serum was quantified fluorometrically using a multimode reader (Synergy H1, Agilent technologies, Santa Clara, CA) at an emission wavelength of 528 nm and an excitation wavelength of 485 nm. All concentrations were measured against a standard curve of serially diluted FITC-dextran.^44^

### Bacterial translocation assay

To assess bacterial translocation following PBI/BM5 (12.5 Gy), mesenteric lymph nodes (MLN) were aseptically collected from the sacrificed mice of the test groups. Collected MLN tissues (1 mg) were homogenized in 1 ml PBS. Homogenates were plated on MacConkey agar plates (BD DIFCO, Franklin Lakes, NJ) and incubated at 37°C for 24–48 h. Colony forming units (CFU) were counted, and densities calculated as follows: CFU/mL = (number of colonies × sample dilution factor × serial dilution factor)/volume of culture plate (mL).^45,46^

### Cytokine analysis

Cytokine quantification in blood serum and colonic tissue samples was performed using the UPLEX assay from Meso Scale Discovery multiplex arrays (MSD, Rockville, MD). Serum samples were diluted two-fold according to the manufacturer’s instructions, and cytokine concentration levels were reported in pg/mL of serum. Cytokine levels in colonic tissue were expressed as pg/100 mg of tissue.^47^ Briefly, flash-frozen colonic samples were homogenized in ice-cold cell lysis buffer (50 mM Tris, pH 7.4, 250 mM NaCl, 5 mM EDTA, 50 mM NaF, 1 mM Na_3_VO_4_, 1% NP40, 0.02% NaN_3_) containing 2X protease inhibitor, using three 40-s bursts using Mini Bead beater 16 (BioSpec Products, Bartlesville, OK). The homogenate was then centrifuged at 14,000 rpm for 10 min at 4°C. The supernatant was collected and used for the assay after determining protein concentrations using the BCA assay method (Pierce, Thermo Fisher Waltham, WA).

### Microbiome analysis

Stool from the lumen and colonic tissue samples from mice were collected, flushed with PBS, frozen in liquid nitrogen, and stored at −80°C. Samples were sent to Zymo Research (Irvine, CA) on dry ice for DNA extraction, library preparation, and sequencing. DNA extraction was performed using the ZymoBIOMICS DNA Miniprep kit and targeted sequencing was prepared with the Quick-16S Plus NGS Library Prep Kit (V3-V4 Primer Set). ZymoBIOMICS Microbial Community DNA Standard was used as a positive control. Real-time PCR was employed to control cycles and minimize PCR chimera formation. Final PCR products were quantified by qPCR, pooled by equal molarity, and cleaned with Select-a-Size DNA Clean & Concentrator. The library was quantified with TapeStation (Agilent Technologies) and Invitrogen Qubit 1X dsDNA HighSensitivity Assay Kits® (Thermo Fisher Scientific, Waltham, WA) and sequenced on Illumina NextSeq 2000 (600 cycles) with 30% PhiX spike-in. DADA2 pipeline was used to infer unique amplicon sequences and remove chimeras.^48^ Taxonomy assignment was performed using UCLUST from QIIME v1.9.1, with biom files analyzed in Microbiome Analyst 2.0^49^ using default parameters: minimum count of 4, 20% prevalence, inter-quantile range for low variance filter, total sum scaling for normalization, and no rarefaction or transformation. α-diversity was assessed using Chao1 and Shannon indexes, and β-diversity via Bray–Curtis dissimilarity with Principal coordinates analysis (PCoA) visualization. Differential abundance was calculated using DEseq2.

### Statistical analysis

Statistical analyses were performed using GraphPad Prism 10. No a priori power analysis was performed to determine sample size. Kaplan–Meier curves, accompanied by log-rank tests, were employed for survival analysis.^32^ For histological analysis and cytokine analysis, comparisons between Vehicle and MIIST305 were made using two-tailed unpaired t-test.^50^ Data are presented as mean ± SEM and results were considered statistically significant at *p* < 5.0E-02. For microbiome analysis, α-diversity was assessed using the Mann–Whitney U test for two groups, and the Kruskal–Wallis multiple comparison test for more than two groups. β-diversity was evaluated with PERMANOVA (Permutational multivariate analysis of variance).^51^ Differential abundance analysis of specific genera was determined by DESeq2 and a *p* < 5.0E-02 (nonparametric test Wilcoxon rank-sum test) was considered significant.

## Results

### MIIST305 increases survival of experimental mice following a lethal dose of acute radiation exposure

**Figure 1.**
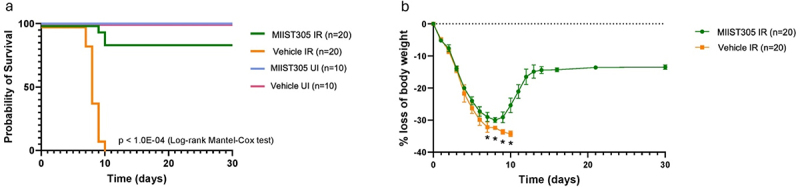
Administration of MIIST305 increases survival and prevents body weight loss in C57BL/6 J mice exposed to 13 Gy PBI/BM5. (a) The Kaplan–Meier curve illustrates the survival of the treatment groups up to 30 DPI. Treatment groups were Vehicle UI (n = 10), MIIST305 UI (n = 10), Vehicle IR (n = 20), and MIIST305 IR (n = 20). Statistical analysis was performed by Logrank (Mantel-Cox) test (b) Comparison of body weight loss (%) following exposure to X-rays at 13.0 Gy, PBI/BM5 measured till 30 DPI (n = 20). Data are presented as mean ± SEM, two-tailed unpaired t-tests were conducted between treatment groups. *p < 5.0E-02 is considered statistically significant with respect to the Vehicle IR.

To evaluate the mitigating efficacy of MIIST305, Vehicle and MIIST305-treated C57BL/6 J male adult mice (n = 20/group) were exposed to 13 Gy of PBI/BM5, and survival was determined up to 30 d post-irradiation. Unirradiated mice (n = 10/group) treated with MIIST305, or Vehicle were used as controls. Exposing Vehicle-treated mice to 13 Gy X-rays was lethal to 100% of Vehicle-treated mice by day 10 following irradiation, whereas approximately 85% (*p* < 1.0E-04) of MIIST305-treated animals survived 30 d post-irradiation (Figure 1a). In both Vehicle- and MIIST305-treated cohorts, animal death occurred between days 7 and 11, consistent with the GIARS time course. All unirradiated mice survived until the end of the experiment on day 30. Both Vehicle and MIIST305-treated animals lost weight in response to 13 Gy X-rays at comparable rates for the first 6 d (Figure 1b). After that, the weight loss of the MIIST305 irradiated mice was significantly less (p < 5.0E-02) compared with Vehicle-treated animals, reaching a nadir of approximately 30% on day 8 and recovery starting from day 9 onwards. By day 14, the MIIST305 mice had regained on average 86% of their pre-irradiation weight that was maintained until the end of the 30-day study period. In contrast, the Vehicle-treated irradiated mice continued to lose weight, and by day 10, all surviving animals had to be euthanized either due to excessive weight loss (35%) or displaying signs of morbidity.

### Administration of MIIST305 improves radiation-induced enteropathy

To analyze temporal colonic structural and functional changes, systemic and local inflammation, as well as gut microbiome changes following high-dose irradiation, we reduced the radiation dose to ensure survival of more of the Vehicle-treated animals at later time points after day 10. At a dose of 12.5 Gy PBI/BM5, we observed that 30% of Vehicle-treated animals survived past day 21 (Figure S1) and therefore, this radiation dose was used to conduct our subsequent studies.

**Figure 2.**
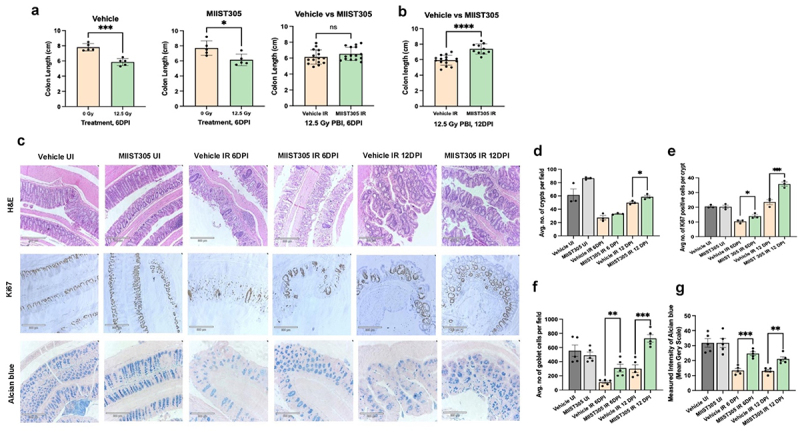
MIIST305 prevents X-ray-induced colon structural and functional damage in C57BL/6 J mice at 12 DPI. (a) Colon lengths from treatment groups were measured at different time points: Vehicle UI (n = 5) vs. Vehicle irradiated IR 6 DPI (n = 5); MIIST305 UI (n = 5) vs. MIIST305 IR 6 DPI (n = 5); Vehicle IR 6 DPI (n = 5) vs. MIIST305 IR 6 DPI (n = 15) (b). Vehicle IR 12 DPI (n = 15) vs. MIIST305 IR 12 DPI (n = 10). (c) histological analysis of colon cross-sections using H&E staining, Ki67 immunohistochemistry, and Alcian blue staining, (10x objective, Scale bar: 50 µm), (d) average number of crypts per field (n = 3), (e) average number of Ki67-positive cells per crypt (n = 3), (f) average number of goblet cells per field (n = 5), (g) mean grayscale intensity of Alcian blue staining (n = 5). Comparisons between treatment groups were performed using two-tailed unpaired t-tests, and the data are presented as mean ± SEM. For colon length, statistical significance is indicated as *p < 5.0E-02, with respect to MIIST305 0 Gy, ***p < 5.0E-04, with respect to Vehicle 0 Gy, ****p < 5.0E-05, with respect to Vehicle IR. For Histological analysis, the statistical significance is indicated as *p < 5.0E-02, **p < 5.0E-03 and ***p < 5.0E-04, with respect to Vehicle IR (6 DPI/ 12 DPI).

Radiation exposure resulted in significant colon length shortening in both Vehicle- and MIIST305-treated animals at 6 DPI, which is a sign of severe tissue injury. Figure 2a depicts the mean colon length of Vehicle UI mice (n = 5) was 7.8 ± 0.21 cm, which was significantly reduced by 24.87 ± 2.70% (p = 2.0E-04) in the Vehicle IR mice (n = 5) at 6 DPI. The MIIST305 IR mice (n = 5) also exhibited a significant reduction in colon length at 6 DPI by 20.25 ± 3.41% (p = 2.0E-02) compared to the MIIST305 UI. The colon length between the irradiated Vehicle- and MIIST305treated mice (n = 15) was similar at 6 DPI with a mean length of 6.14 ± 0.23 cm and 6.53 ± 0.21 cm, respectively. However, by 12 DPI, the mean colon length of the MIIST305 IR mice (n = 10) were found to be 7.37 ± 0.20 cm, which was significantly longer by 19.67 ± 2.23% (p = 1.0E-04) compared to the Vehicle IR mice (n = 15) (Figure 2b).

Histopathological examination demonstrated loss of colonic crypts on 6 DPI with a partial recovery on 12 DPI that was higher in the MIIST305-treated mice compared to the Vehicle-only mice (Figure 2c and 2d). The average number of crypts, in the tissue sections of the Vehicle UI and MIIST305 UI group were 61.33 ± 9.03 and 86.46 ± 1.07, respectively, which was reduced by 55.22 ± 1.47% and 61.70 ± 0.78% in Vehicle IR and MIIST305 IR at 6 DPI with respect to Vehicle UI and MIIST305 UI. MIIST305 treatment did not have any significant impact on the number of crypts at 6 DPI, but at 12 DPI an increase in the average number of crypts was observed in MIIST305-treated mice by 14.91 ± 2.60% (p = 3.5E-02) with respect to the Vehicle-only mice. The average number of proliferative cells/crypts as determined by Ki67 immunostaining was significantly higher in the MIIST305-treated group by 25.42 ± 5.27% (p = 4.0E-02) at 6 DPI and by 34.28 ± 4.71% (p = 3.0E-02) at 12 DPI compared to the Vehicle-only treated group as represented in Figure 2e.

Loss of goblet cells and dysregulation of mucus production and secretion has been associated with intestinal epithelial barrier dysfunction, microbiome dysbiosis, increased inflammation and infectious diseases. Staining with the goblet cell marker Alcian blue (stains acidic mucins and mucopolysaccharides) demonstrated that mice treated with MIIST305 had a significantly higher number of goblet cells compared with Vehicle-treated mice on 6 DPI and 12 DPI (Figure 2c and 2f). The average number of goblet cells per field in the Vehicle UI and MIIST305 UI groups were 554.64 ± 79.94 and 491.08 ± 44.18, respectively, (Figure 2c and 2f).

These numbers were significantly reduced by 80.17 ± 3.12% (p = 6.0E-04) and 36.78 ± 10.80% (p = 3.0E-02) following 12.5 Gy PBI/BM5 exposure in the Vehicle IR and MIIST305 IR groups, respectively, at 6 DPI (Figure 2f). However, at 6 DPI and 12 DPI, the average number of goblet cells was significantly higher in the MIIST305-treated group by 64.48 ± 5.58% (p = 7.1E-03) and 58.65 ± 7.53% (p = 5.0E-04), respectively, compared to the Vehicle IR.

Likewise, mucin production per goblet cells, quantitated by measuring the intensity of the Alcian blue signal per cell, was also significantly higher in MIIST305-treated animals (Figure 2c and 2g). The mean gray scale of Alcian blue staining was highest in the Vehicle UI (31.76 ± 2.79) and MIIST305 UI (31.73 ± 2.98) groups, as represented in Figure 2g. At 6 DPI and 12 DPI, the mean gray scale of MIIST305 IR was significantly higher by 45.53 ± 4.92% (p = 2.0E-04) and 37.88 ± 4.15% (p =1.5E-03) compared to the Vehicle-only treated group.

### MIIST305 reduces intestinal epithelial barrier permeability and bacterial translocation in mice exposed to irradiation

**Figure 3.**
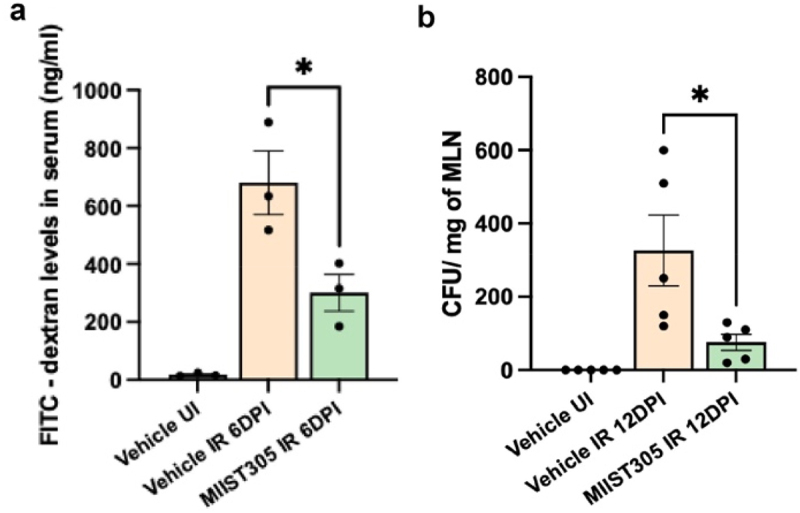
MIIST305 ameliorates gut barrier function. (a) Fluorometric quantification of FITCdextran levels (ng/ml) obtained from serum samples of treatment groups (n = 3), (b) Count of CFU on MacConkey agar to quantify gut bacterial translocation in MLN (n = 5). Data are represented as mean ± SEM, two-tailed unpaired t-tests were conducted between treatment groups, *p < 5.0E02 is considered statistically significant with respect to Vehicle IR (6 DPI/ 12 DPI).

To investigate whether MIIST305 treatment administered at 24 h post-irradiation with additional doses of MIIST305 on days 3 and 5 improves intestinal epithelial barrier function, we performed the FITC-dextran, barrier permeability assay and assessed bacterial translocation from the intestinal lumen to the MLNs on 6 DPI involving animals (n = 3/group) from the Vehicle UI, Vehicle IR and MIIST305 IR experimental groups. As expected, radiation exposure resulted in a large increase in barrier permeability in Vehicle-treated mice compared with unirradiated mice. However, treatment with MIIST305 decreased barrier permeability defects, observed by the lower concentration of FITC-dextran (55.73 ± 9.26%; p = 4.0E-02) in the serum on 6 DPI compared with the Vehicle IR group (Figure 3a).

Bacterial translocation to MLNs indicates a defective gut barrier function. MLNs were collected from Vehicle UI, Vehicle IR and MIIST305 IR groups (n = 5/group) at 12 DPI and obtained result shows that radiation exposure resulted in significant increase in bacterial translocation to MLNs compared with unirradiated mice (Figure 3b). However, treating irradiated mice with MIIST305 significantly inhibited bacterial translocation. Notably, bacterial translocation was significantly reduced in MIIST305-treated mice (76.69 ± 6.69%; p = 3.0E-02) compared to the Vehicle IR group on 12 DPI.

### Oral administration of MIIST305 modulates serum and colonic cytokine levels following radiation exposure

**Figure 4.**
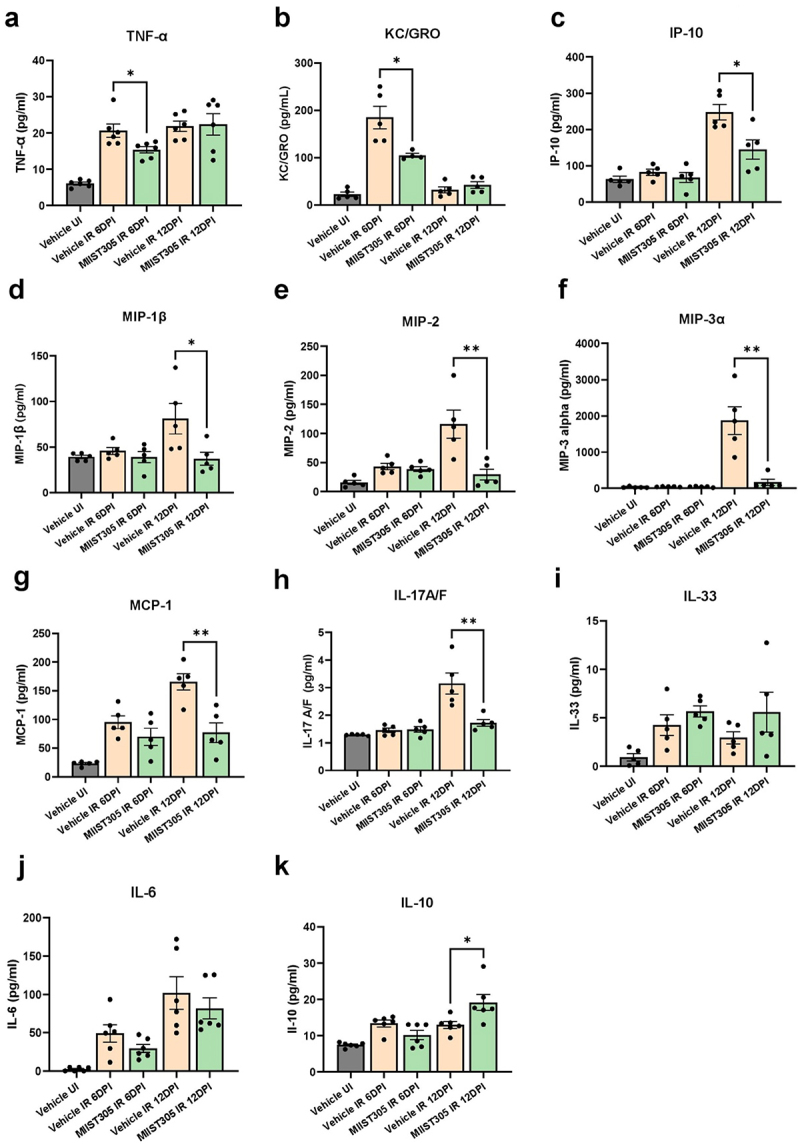
MIIST305 mitigates X-ray-induced inflammation by reducing pro-inflammatory cytokine levels in serum and increasing the anti-inflammatory cytokine IL-10. Quantification of serum cytokines levels (pg/ml) using MSD assay (a) TNF-α, (b) KC/GRO, (c) IP-10, (d) MIP-1β, (e) MIP2, (f) MIP-3α, (g) MCP-1, (h) IL-17A/F, (i) IL-33, (j) IL-6 and (k) IL-10. Data are represented as mean ± SEM, two-tailed unpaired t-tests were conducted between treatment groups, *p < 5.0E02 is considered statistically significant with respect to Vehicle IR (6 DPI/12 DPI), **p < 5.0E-03 is considered statistically significant with respect to Vehicle IR (6 DPI/12 DPI).

It is well documented that radiation exposure elicits a cytokine storm both at the systemic and damaged organ-specific level. Here, we measured the levels of a panel of 29 cytokines in both serum and colonic tissue samples (n = 3–8/group) at 6 DPI and 12 DPI following 12.5 Gy PBI/BM5 X-ray exposure. Radiation increased levels of several pro-inflammatory cytokines in the serum including TNF-α, KC/Gro, IP-10, MIP-1β, MIP-2, MIP-3α, MCP-1, IL-17A/F, IL-33 and IL-6, as well as the anti-inflammatory cytokine IL-10 (Figure 4). Specifically, TNF-α and KC/Gro levels were reduced by 25.47 ± 4.37% (p = 2.0E-02) and 43.45 ± 2.36% (p = 5.0E-02) in the MIIST305 IR mice when compared to the Vehicle-only group at 6 DPI, whereas the expression levels of IP-10, MIP-1β, MIP-2, MIP-3α, MCP-1 and IL-17A/F were significantly lower in the MIIST305 IR group compared to the Vehicle IR group by 41.45 ± 10.65% (p = 5.0E-02), 54.26 ± 8.65% (p = 4.0E02), 74.68 ± 7.98% (p = 9.0E-03), 87.80 ± 4.61% (p = 2.0E-03), 53.49 ± 10.32% (p = 4.0E-03) and 45.22 ± 3.79% (p = 7.0E-03), respectively, at 12 DPI. Additionally, serum levels of IL-6 were reduced and IL-33 were increased with MIIST305 treatment, but no statistical significance was attained. In contrast, the concentration of anti-inflammatory cytokine IL-10, was significantly increased in the MIIST305 IR mice by 32.27 ± 11.59% (p = 2.0E-02) compared to the Vehicle only mice at 12 DPI (Figure 4k). Furthermore, irradiation led to an elevation in serum levels of IL2 (p = 1.0E-02), IFN-γ (p = 1.04–03), IL-17A (p = 3.3E-02), IL-22 (p = 4.1E-02) and IL-27p28/IL30 (p = 1.5E-03) in Vehicle IR group at 12 DPI with respect to Vehicle UI; however, treatment with MIIST305 did not modulate the concentrations of these cytokines (Figure S2). Additionally, no significant changes were observed in the serum levels of IL-1β, MIP-1α, and IL-23 in response to irradiation (Figure S2).

**Figure 5.**
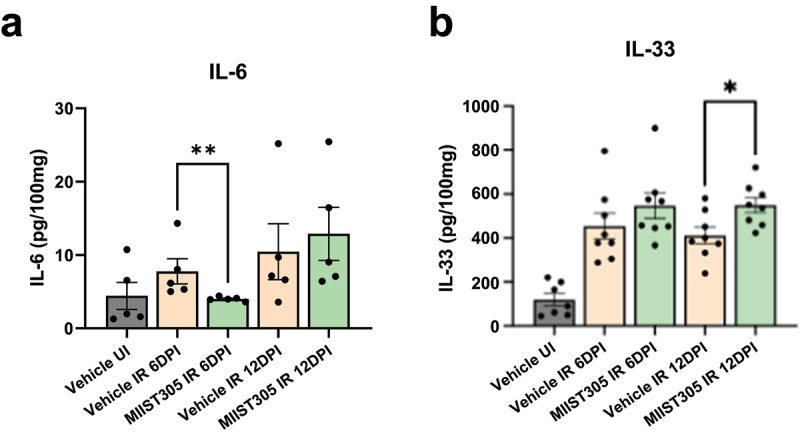
MIIST305 reduces X-ray-induced inflammation by modulating cytokine levels at colonic tissue, specifically decreasing IL-6 and increasing IL-33. Quantification of cytokine levels (pg/100 mg) in colonic tissue was performed using MSD assay for (a) IL-6 (n = 5) and (b) IL-33 (n = 8). Data are presented as mean ± SEM. Two-tailed unpaired t-tests were conducted between treatment groups, with *p < 5.0E-02 considered statistically significant compared to Vehicle IR 12 DPI and **p < 5.0E-03 considered statistically significant compared to Vehicle IR 6 DPI.

In colonic tissue lysates, IL-6 expression was significantly reduced by 48.51 ± 1.70% (p = 7.0E-03) in MIIST305-treated mice compared to the Vehicle-treated at 6 DPI, whereas IL-33 levels in the MIIST305 IR group increased by 25.14% ± 6.97% (p = 1.8E-02) compared to the Vehicle IR group at 12 DPI (Figure 5). Further, the levels of IL-1β (p = 1.2E-02; 12 DPI), IL-2 (p = 5.0E-04; 6 DPI, p = 3.8E-03; 12 DPI), TNF-α (p < 1.0E-04; 6 DPI, p = 7.0E-04; 12 DPI), KC/Gro (p = 7.0E-04; 6 DPI, 3.0E-04; 12 DPI), IFN-γ (p = 2.5E-03; 6 DPI, p = 1.8E-03; 12 DPI), IL-10 (p = 3.8E-02; 12 DPI), IL-17A (p < 1.0E-04; 6 DPI, p = 1.6E-03; 12 DPI), IL-22 (p = 2.0E-04; 6 DPI, p = 2.7E-03; 12 DPI), IL-23 (p = 4.1E-02; 6 DPI, p = 1.7E-02; 12 DPI), IL-27p28/IL-30 (p = 4.8E02; 6DPI; p = 1.5E-03; 12 DPI), IP-10 (p = 3.0E-04; 6 DPI, p = 5.5E-03; 12DPI), MIP-1β (p = 3.9E02; 6 DPI), MIP-2 (p = 8.6E-03; 6 DPI, p = 3.7E-02), MIP-3α (p = 2.0E-03; 6 DPI) and MCP-1 (p < 1.0E-04; 6 DPI, p = 1.0E-04; 12 DPI) were elevated in tissue lysates following irradiation (Vehicle IR) on either 6 DPI/12 DPI or both time points with respect to Vehicle UI in colonic tissues, but treatment with MIIST305 did not influence any of these cytokine levels. Furthermore, no changes in IL-17 A/F and MIP-1α levels were observed in response to irradiation (Figure S3).

### MIIST305 restores a more normal microbiome distribution in GI injury

Gut microbiota reinforces the intestinal epithelial barrier function and reduces inflammation.^52^ However, radiation exposure triggers intestinal microbiome dysbiosis affecting the diversity and composition of microbiota that leads to intestinal epithelial cell barrier damage and local and systemic inflammation.^53^ To investigate the effect of 12.5 Gy X-ray PBI/BM5 on the gut microbiome of C57BL/6 J mice, 16S rRNA amplicon sequencing was conducted on mucosal and luminal samples from the Vehicle- and MIIST305-treated IR mice, analyzed at 6 and 12 DPI. Comparative analysis was performed against unirradiated controls (Vehicle UI and MIIST305 UI).

**Figure 6.**
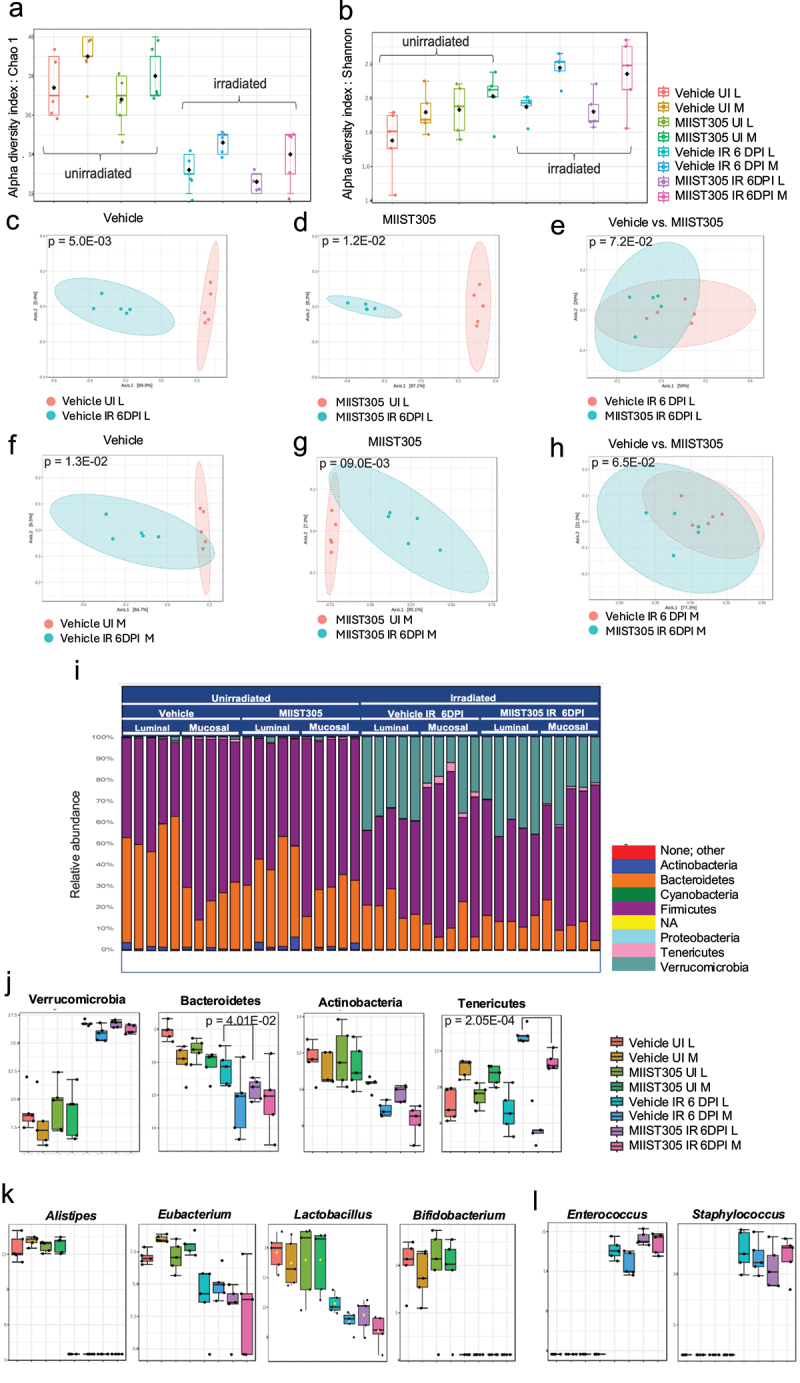
X-ray induces gut dysbiosis and alters gut microbial composition in C57BL/6 J (n = 5) mice at 6 DPI. Representation of (a) Chao index. (b) Shannon index. PCoA analysis of (c) Vehicle UI L vs Vehicle IR 6 DPI L, (d) MIIST305 UI L vs MIIST305 IR 6 DPI L, (e) Vehicle IR 6 DPI L vs MIIST305 IR 6 DPI L (f) Vehicle UI M vs Vehicle IR 6 DPI M, (g) MIIST305 UI M vs MIIST305 IR 6 DPI M (h) Vehicle IR 6 DPI M vs MIIST305 6 DPI M, (i) the relative abundance at phylum level, (j) differential abundance of bacteria at phylum level (k) differential abundance of bacteria at genus levels. L and M denotes luminal and mucosal, respectively. For α-diversity (Chao1 and Shannon index) and β-diversity (PCoA plots) analysis, comparison between test groups was conducted using the Mann–Whitney U test and the PERMANOVA test respectively. For relative abundance at phylum level and differential abundance at genus levels, comparisons between treatment groups were performed using two-tailed unpaired t tests, the data are presented as mean ± SEM and p < 5.0E-02 is considered statistically significant. UI: unirradiated; IR: irradiated; L: luminal; M: mucosal; DPI: days post-irradiation.

Radiation exposure resulted in a significant reduction in α-diversity (p < 5.0E-02) at the genus rank as judged by the Chao1 index, which accounts for taxa richness within a group, in both the luminal and the mucosal compartment on day 6 post-irradiation (Figure 6a). Although not statistically significant, mucosal microbiota showed greater α-diversity compared with luminal microbiota. MIIST305 treatment did not have an influence on α-diversity. On the other hand, irradiated and unirradiated groups displayed similar microbiota diversity as measured by the Shannon index, which takes into consideration the richness and evenness of taxa within a group (Figure 6b). Again, mucosal samples displayed higher, but not statistically significant values compared with luminal samples in both unirradiated and irradiated cohorts. PCoA visualization of β-diversity (Figure 6c–6h) at the genus level using the Bray–Curtis dissimilarity metric revealed a clear distinction in microbial diversity between Vehicle UI and Vehicle IR 6 DPI in luminal (p = 5.0E-03) and mucosal (p = 1.3E-02) samples. Similar results were also observed with luminal (p = 1.2E-02) and mucosal (p = 9.0E-03) samples of MIIST305 UI and MIIST305 IR 6 DPI. However, at 6 DPI the microbiome profiles of Vehicle IR and MIIST305 IR were closely related for both luminal (p = 7.2E-02) and mucosal (p = 6.5E-02) samples highlighting their similar composition.

Taxonomic profiling at the phylum level (Figure 6i) showed that the gut bacterial microbiome from unirradiated mice was dominated by Firmicutes and Bacteroidetes, whereas low levels of Actinobacteria, Tenericutes, and Verrucomicrobia were also detected. The taxonomic profile and relative abundance of MIIST305-treated unirradiated mouse microbiota was very similar to that of Vehicle-treated unirradiated animals. However, statistically significant differences in the relative abundance of phyla were revealed between luminal and mucosal compartments. Specifically, the relative abundance of Bacteroidetes was 52.54 ± 3.25% in the lumen and 24.30 ± 3.07% in the mucosa (p = 2.3E-04) for the Vehicle-treated animals and 39.94 ± 3.59% in the lumen and 26.90 ± 3.13% in the mucosa (p = 2.5E-02) for the MIIST305-treated mice. The relative abundance of Firmicutes was 44.94% ± 3.35% (luminal), 73.62 ± 3.25% (mucosal) for the Vehicle mice (p = 2.8E-04) and 56.18 ± 3.90% (luminal), 70.24 ± 3.44% (mucosal) for the MIIST305 mice (p = 2.7E-02). Tenericutes also showed differential abundance under basal conditions, displaying significantly higher relative abundance (p = 1.4E-02 Vehicle; p = 4.9E-02 MIIST305) in the mucosa compartment (0.92 ± 0.15% Vehicle; 0.74 ± 0.12% MIIST305) compared with luminal compartment (0.38 ± 0.09% Vehicle; 0.42 ± 0.07% MIIST305). Lastly, Actinobacteria and Verrucomicrobia were equally distributed in the two colonic compartments.

Radiation exposure led to significant changes in the abundance of several phyla (Figure 6i and 6j). Microbiome differential abundance analysis on day 6 post-irradiation revealed a dramatic increase in the abundance of Verrucomicrobia. In particular, samples from Vehicle IR exhibited a relative abundance of 38.24 ± 1.69% in the luminal compartment and 22.72 ± 3.92% in the mucosal compartment at 6 DPI. In contrast, in Vehicle UI samples, the relative abundance of Verrucomicrobia was markedly lower, with values of 0.48 ± 0.35% in the luminal and 0.22 ± 0.19% in the mucosal compartments. A similar increase was also noted in MIIST305-treated groups at 6 DPI, with 40.56 ± 3.16% in luminal samples and 28.10 ± 3.67% in mucosal compartments with respect to MIIST305 UI. Bacteroidetes and Actinobacteria were significantly reduced in both the luminal and mucosal compartment (Figure 6i and 6j). Firmicutes were also reduced in response to irradiation; however, this reduction was not statistically significant. Interestingly, Tenericutes displayed a differential response to radiation at 6 DPI depending on the treatment and the localization of the bacteria. Specifically, radiation exposure resulted in a significant increase of mucosal Tenericutes in the Vehicle (p = 2.7E-05), but not MIIST305 (p = 9.3E-02) cohorts, whereas luminal Tenericutes abundance increased in the MIIST305-treated (p = 1.4E-02), but not Vehicle-treated (p = 9.6E-01) animals (Figure 6j and S4a). However, the difference in Tenericutes abundance between Vehicle and MIIST305 was significantly only in the mucosal (63.84 ± 6.25%; p = 2.1E-04), but not luminal (p = 1.7E-01) compartment. Taxonomic analysis at the genus levels revealed that *Anaeroplasma* (Figure S4b) accounted entirely for the relative abundance of Tenericutes. Differential abundance analysis of the microbiome at the genus level revealed a reduction in a number of beneficial bacteria such as *Alistipes, Eubacterium, Lactobacillus*, and *Bifidobacterium* (Figure 6k) and an increase of the potentially pathogenic bacteria *Enterococcus* and *Staphylococcus* (Figure 6l) in both the luminal and mucosal samples of irradiated animals compared with unirradiated animals, whereas MIIST305 treatment did not have an impact on these microbial shifts at 6 DPI.

**Figure 7.**
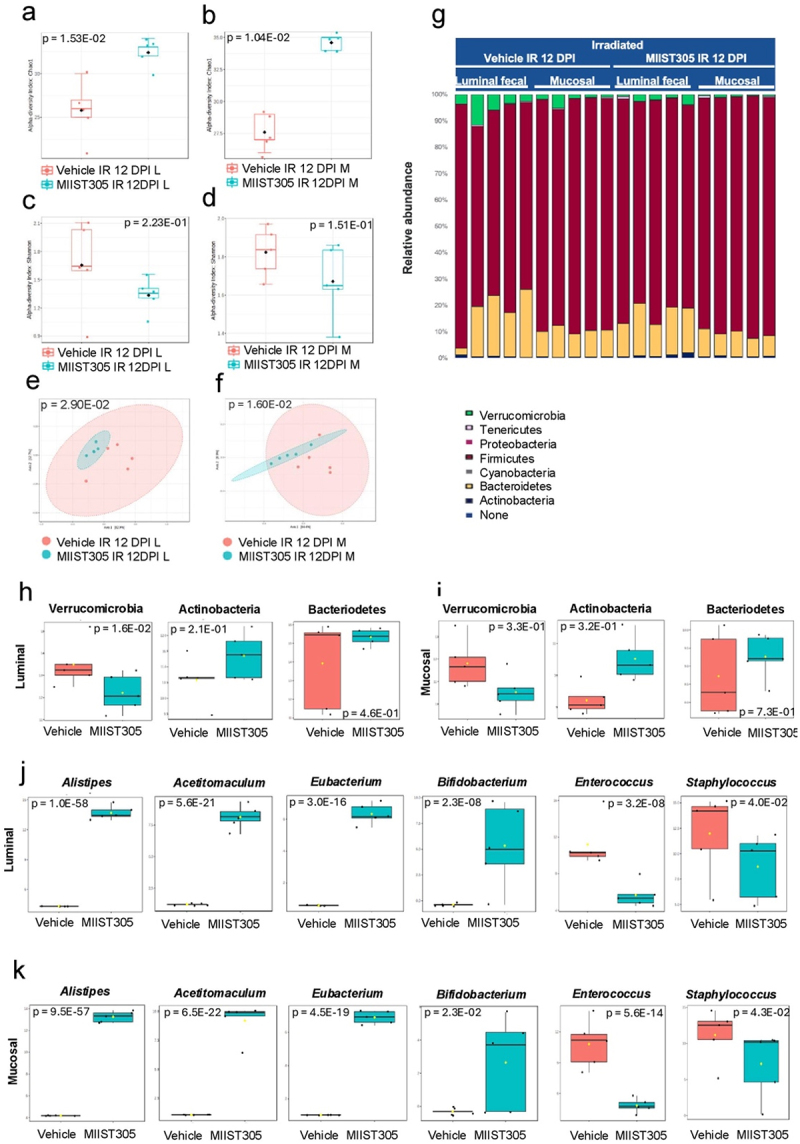
MIIST305 reduces x -ray induced gut dysbiosis in C57BL/6 J (n = 5) mice at 12 DPI. Representation of Chao1 index (a) luminal, (b) mucosal, Shannon index (c) luminal, (d) mucosal of the intestinal microbiota between Vehicle IR 12 DPI and MIIST305 IR 12 DPI. PCoA score plots (Bray – Curtis) between Vehicle and MIIST305 at genus level (e) Luminal (f) Mucosal (g) relative abundance at phylum level, differential abundance of bacteria at phylum level (h) luminal (i) mucosal, differential abundance of bacterial genera (j) Luminal (k) Mucosal, L and M denotes luminal and mucosal, respectively. For α-diversity (Chao1 and Shannon index) and β-diversity(PCoA plots) analysis, comparison between test groups was conducted using the Mann–Whitney U test and the PERMANOVA test respectively. For relative abundance at phylum level and differential abundance at genus levels, comparisons between treatment groups were performed using two-tailed unpaired t tests, the data are presented as mean ± SEM and p < 5.0E-02 is considered statistically significant. UI: unirradiated; IR: irradiated; L: luminal; M: mucosal; DPI: days post-irradiation.

Next, we compared the microbiome diversity and composition of Vehicle IR and MIIST305 IR samples on 12 DPI. α-diversity using the Chao1 index showed that MIIST305 IR genera richness was significantly higher in both luminal (p = 1.5E-02) and mucosal (p = 1.0E-02) samples compared with Vehicle IR samples (Figures 7a and 7b). In contrast, Shannon index did not show appreciable differences between the two groups (Figures 7c and 7d). β-diversity examination using the Bray–Curtis dissimilarity test and visualized by PCoA showed that MIIST305 IR samples clustered separately from Vehicle IR samples in both lumen and mucosa (Figures 7e and 7f), implying the two groups had significantly different bacterial composition. The relative abundance at the phylum rank showed that Firmicutes abundance had mostly recovered from 6 DPI and almost completely dominated the phylum taxa, whereas Bacteriodetes remained at low levels (Figure 7g). By 12 DPI, there was a significant reduction in Verrucomicrobia abundance in both luminal and mucosal samples, especially in the MIIST305 IR samples (Figures 7g,h,i). Among differentially abundant genera, the presence of *Alistipes, Acetitomaculum, Eubacterium* and *Bifidobacterium* were highly enriched in the luminal and mucosal compartment of MIIST305 IR samples with respect to Vehicle IR. In contrast, the abundance of *Enterococcus* and *Staphylococcus* in luminal and mucosal compartments of MIIST305 samples were significantly reduced compared with Vehicle IR (Figures 7j and 7k). Finally, a composition analysis at the species level revealed that *Lactobacillus johnsonii* abundance accounted for the majority of species (Figures S4c and S4d) and it was more abundant in the MIIST305 IR samples compared with Vehicle IR samples. The relative abundance of *L. johnsonii* at 12 DPI was 33.68 ± 12.36% and 64.28 ± 3.05% (p = 4.3E-02) in the luminal compartment and 16.72 ± 5.90% and 42.26 ± 7.16% (p = 2.5E-02) for the mucosal compartment of Vehicle IR and MIIST305 IR groups, respectively.

## Discussion

As a rapidly self-renewing tissue, the GI epithelium is particularly sensitive to ionizing radiation.^54–57^ Radiation-induced intestinal epithelial cell death results in the loss of GI epithelial integrity and function characterized by epithelial erosion,^58^ crypt distortion^59^ and goblet cell depletion.^60^ These changes compromise the gut barrier allowing luminal antigens and microorganisms to penetrate the lamina propria and enter the bloodstream through the MLN. This breach triggers a strong local and systemic pro-inflammatory response, which further exacerbates damage to the gut.^61^

Our results show that MIIST305 exhibits strong radio-mitigating effects in mice, when administered 24 h after a single high dose of partial body irradiation that otherwise would be lethal due to GI injury^62,63^ (Figure 1). Whereas radiation exposure diminished the number of proliferating epithelial cells in the colonic crypts^64^ and reduced goblet cells,^65^ MIIST305 treatment maintained increased numbers of proliferating cells per colonic crypt, as well as increased numbers of goblet cells per crypt and higher production of mucins per cell, on both days 6 and 12 post-irradiation (Figure 2). These results suggest that MIIST305 could play an important role in restoring the integrity of the mucus layer after radiation exposure, thereby reinforcing gut barrier function. Concordantly, we show that MIIST305 treatment reduced gut barrier permeability and greatly diminished bacterial translocation to MLN (Figure 3).

It is well established that radiation exposure can elicit an inflammatory response that contributes to multi-organ injury.^66^ Pro-inflammatory cytokines such as IL-1β, IL-6, and TNFα play major roles in the development of radiation-induced intestinal damage.^12,67^ In the present study we undertook a comprehensive evaluation of several GI injury-related pro- and anti-inflammatory cytokines in the serum (Figure 4) and colon lysates (Figure 5). The majority of cytokines, including IL-1 β, TNFα and IL-6, were induced both systemically and in the colon in response to radiation. Our findings also demonstrate that cytokines like IL-17A and IL-22, which are well recognized for their roles in intestinal pathophysiology, are also upregulated in response to radiation. Most of the pro-inflammatory cytokines (e.g., MIP-1β, MIP-2, MIP-3α, and MCP-1) that were upregulated by radiation were related to macrophage function with roles in trafficking immune cells.^68–71^ MIIST305 treatment resulted in the reduction of many pro-inflammatory cytokines in serum. With the exception of TNFα and KC/GRO that were suppressed on day 6 post-irradiation in the MIIST305treated mice, all other cytokines showed diminished levels on day 12 post-irradiation, suggesting MIIST305 has a significant role in suppressing radiation-induced inflammation. The anti-inflammatory cytokine IL-10^72^ was significantly upregulated in the serum of the MIIST305-treated mice compared with Vehicle-treated mice (Figure 4). In contrast to its effects on circulation, MIIST305 exhibited a relatively limited influence on cytokine levels within colonic tissue. We demonstrate that MIIST305 transiently reduced levels of IL-6 and increased levels of IL-33 in the colon (Figure 5). IL-6 exerts a dual role on GI physiology, which is modulated by the composition of gut microbiota. During bacterial dysbiosis, IL-6 has been demonstrated to promote inflammatory responses and tissue damage.^73^ IL-33 is an alarmin cytokine that is released upon tissue damage,^74^ and it has been shown to restore barrier integrity by promoting IEC regeneration after intestinal injury as well as increase goblet cell numbers and Muc2 production.^75^ Furthermore, IL-33 promotes an immunosuppressive environment in the presence of IL-10 by inducing regulatory T cell function in the intestine.^76^ Recently, it has been shown that IL-33 potentiates intestinal stem cell regeneration in ileum after radiation injury through a mechanism that involves secretion of Epidermal Growth Factor by Paneth cells.^77^ We show here that radiation exposure results in a robust induction of IL-33 in the mouse colon as well as that MIIST305 upregulates its expression. Considering that Paneth cells are not normally found in colon, it would be of interest to elucidate the mechanism of colonic IL-33 induction in response to irradiation.

We explored the impact of radiation on the gut microbiome community in both the luminal and colonic mucosal compartment in mice treated with MIIST305 or Vehicle. In the human gut, it has been demonstrated that the luminal microbiota and the mucosa-associated microbiota differ in diversity and composition.^78,79^ Radiation exposure reduced α-diversity (Chao1 index) on day 6 post-irradiation, whereas irradiated and unirradiated samples were clustered separately (βdiversity) suggesting the two bacterial communities were significantly different from each other. MIIST305 treatment did not have a significant effect on α-diversity at day 6 (Figure 6). However, by day 12, the MIIST305-treated mice indicated greater α-diversity compared with Vehicle-treated animals. β-diversity analysis demonstrated a clear separation between unirradiated and irradiated microbiota groups in both the lumen and the mucosa at day 6 in the Vehicle and MIIST305-treated mice on day 12, suggesting the two groups had divergent microbial composition.

Taxonomic profiling of gut microbiota at the phylum level revealed that radiation exposure caused a large increase in the relative abundance of Verrucomicrobia (represented by the species *Akkermansia muciniphila*) at day 6, which is consistent with previous reports;^80–82^ however we show here that this increase was transient and greatly diminished by day 12 post-irradiation, especially in the MIIST305-treated animals that returned to pre-irradiation levels (Figure 7). *A. muciniphila* is a probiotic under homeostatic conditions and has been associated with several beneficial effects,^83^ but it has also been associated with adverse effects in some circumstances. For example, increased levels of *A. muciniphila* have been reported in colitis patients, and it is negatively associated with survival after total body irradiation.^84^

Besides Verrucomicrobia, the relative abundance of Bacteroidetes and Actinobacteria was reduced following irradiation in both the lumen and colonic mucosa, similar to previous findings. On the other hand, we show for the first time that the abundance of Tenericutes (genus *Anaeroplasma*) in the colonic mucosa increased following radiation exposure, but only in the Vehicle-treated animals, whereas MIIST305-treated animals showed a decrease in the luminal compartment on 6 DPI. However, by 12 DPI, Tenericutes had returned to pre-irradiation levels for both treatment groups. Previous studies have reported that elevated levels of Tenericutes are associated with chronic gut inflammation and are directly linked to increased expression of IL-6 in the colonic environment.^85^ Interestingly, we show that MIIST305-treated animals display a significantly reduced concentration in colonic IL-6 levels at 6 DPI, consistent with the diminished levels of mucosal Tenericutes compared with Vehicle-treated animals.

A taxonomic examination at the genus levels revealed that a number of beneficial bacterial genera were driving the differences in bacterial community on day 6 after irradiation between unirradiated and irradiated animals. Specifically, *Alistipes, Bifidobacterium, Eubacterium*, and *Lactobacillus* levels were severely reduced compared with unirradiated mice (Figure 6). *Alistipes* has been suggested to have a protective role against colitis and has been found to be suppressed in response to radiation and chemotherapy.^79^ *Bifidobacterium spp*. are known probiotics that increase production of Muc2 and tight junction proteins, thus, decreasing intestinal permeability and reducing bacterial translocation. In relation to human disease, *Bifidobacterium spp*. have been shown to downregulate Toll-like receptor 4 (TLR4)/NF-κB signaling in IECs and prevent acute colitis. *Eubacterium spp*. are major butyrate producers in the gut that have beneficial impact on colonocytes and the intestinal epithelial barrier, and they suppress TNFα-induced TLR4/NF-κB axis and intestinal inflammation.^86^ They also produce valerate that has been shown to confer radioresistance in animal models.^13^ On the other hand, while the genera *Enterococcus* and *Staphylococcus*, which include several potentially pathogenic species, were nearly undetectable in animals under homeostatic conditions, radiation exposure led to a marked increase in their abundance. The data show that MIIST305 treatment did not impact significantly the differential abundance of these genera on 6 DPI. However, at 12 DPIMIIST305 treatment induced notable shifts in several genera. Specifically, MIIST305-treated mice exhibited a pronounced increase in *Alistipes, Acetatimaculum, Eubacterium*, and *Bifidobacterium* compared to Vehicle-treated mice, whereas *Enterococcus* and *Staphylococcus* levels were significantly reduced in MIIST305-treated mice, albeit remained elevated compared to unirradiated controls (Figure 7).

One interesting finding of the current study is the predominance of the species *Lactobacillus johnsonii* (*L. johnsonii*) in the recovering colon following radiation exposure. *L. johnsonii* abundance was greatly diminished on day 6 following radiation exposure. However, on day 12, *L. johnsonii* abundance was dramatically enhanced (mainly in the MIIST305-treated animals) accounting for approximately 64% and 42% of all bacterial species in the luminal and mucosal compartment of MIIST305-treated samples (Supplementary Figure 4). *L. johnsonii* is a known probiotic and several strains are under evaluation against several human diseases.^87^ *L. johnsonii* has been shown to ameliorate oxidative stress, systemic genotoxicity,^80^ and alleviate the damage caused by microbial pathogens by inhibiting TLR4, NF-κB, and the inflammasome (NLRP3) signaling pathway.^88^ *L. johnsonii* promotes gut barrier homeostasis^89^ and inhibits several pathobionts from adhering to the colonic mucosal,^90^ thus preserving gut barrier function and suppressing inflammation. Furthermore, *L. johnsonii* alone or in cooperation with other bacterial species is implicated in the synthesis of a number of metabolites that are critical in maintaining host GI health.^87,91^

In summary, we show that MIIST305 has significant potential as an effective mitigator of GI-ARS. MIIST305 enhances the survival rate of irradiated mice by preserving the intestinal barrier function, reducing bacterial translocation, suppressing radiation-induced inflammatory cytokine response, ultimately facilitating the recovery of the structure and function of the intestine. Furthermore, MIIST305 reduces systemic inflammation in response to radiation suggesting that the drug could alleviate the risk of detrimental immune-mediated organ damage. Whereas radiation exposure leads to bacterial microbial imbalance (dysbiosis), which is characterized by the loss of beneficial species and the overgrowth of pathobionts, MIIST305 decreases potential pathobionts while expanding commensal protective bacteria. Future studies are directed toward determining the mechanism of action of MIIST305, as well as providing the mechanistic relationship between microbiota and radiation-induced bowel injury. Correlation studies between gut microbiota composition, histopathological alterations, and cytokine modulation may enlighten specific microbial taxa with a causative impact on GI-ARS. Furthermore, elucidating significantly changed circulating and tissue cytokines after partial body irradiation will aid in deciphering the mechanism of GI-ARS and identifying potential biomarkers to predict clinical outcome. The experimental studies performed here used only male mice. Previous studies have documented sex-specific differences in gut microbiota composition under basal conditions and in response to acute high-dose radiation^92,93^ and the systemic and intestinal mucosal immune system.^89^ In future work, we intend to evaluate MIIST305 radiomitigating efficacy on survival and immune cell response in female mice.

## Abbreviations

BM5: 5% bone marrow shielding
CFU: Colony forming units
CUIMC: Columbia University Irving Medical Center
DPI: days post irradiation
FDA: U.S. Food and Drug Administration
FMT: fecal microbiota transplant
GI: gastrointestinal
IECs: intestinal epithelial cells
MCMs: medical countermeasures
MIIST: multivalent innate immune signaling target platform
PBI: partial body irradiation
SSD: source to surface distance
IACUC: Institutional Animal Care and Use Committee
UI: unirradiated
IR: irradiated
MISS: Mouse Interventional Scoring System
MLNs: mesenteric lymph nodes
PBS: phosphate-buffered saline
PcoA: Principal coordinates analysis
PERMANOVA: Permutational multivariate analysis of variance
WBC: white blood cells

## Supplementary Material

Supplemental Material

## Data Availability

All the generated data from the study are either embedded in the main text file or in the supplementary files, any further details will be made available on request. The raw data generated from the microbiome analysis is deposited into NCBI database with reference “PRJNA1176683” and will be available upon publication.
