# Supplementary material for: MIIST305 mitigates gastrointestinal acute radiation syndrome injury and ameliorates radiation-induced gut microbiome dysbiosis
Source: Gut Microbes. 2025 Feb 10;17(1):2458189. doi: 10.1080/19490976.2025.2458189 (PMC11817531; doi:10.1080/19490976.2025.2458189)
Supplement: Supplemental Material [file KGMI_A_2458189_SM1171.docx]

Sl


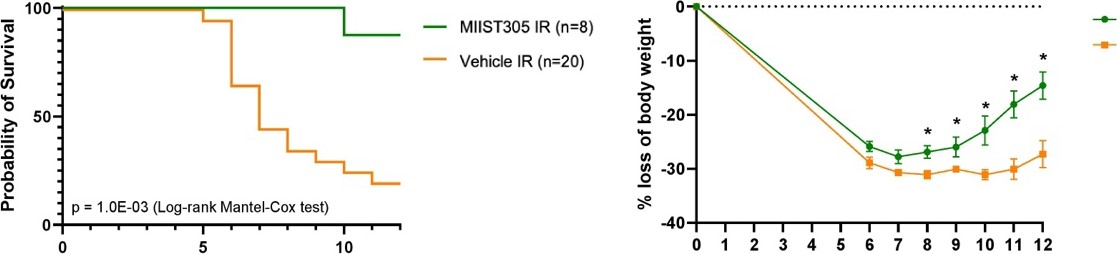
MllST305 IR (n=8)

Vehicle IR (n=20)

Time (days) Time (days)


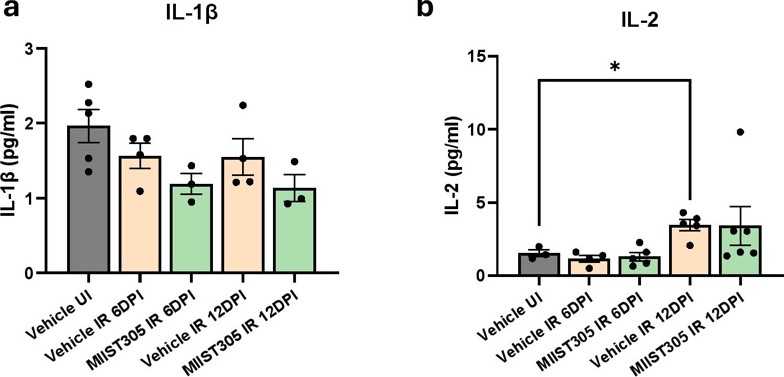


M旧-10 に-17A


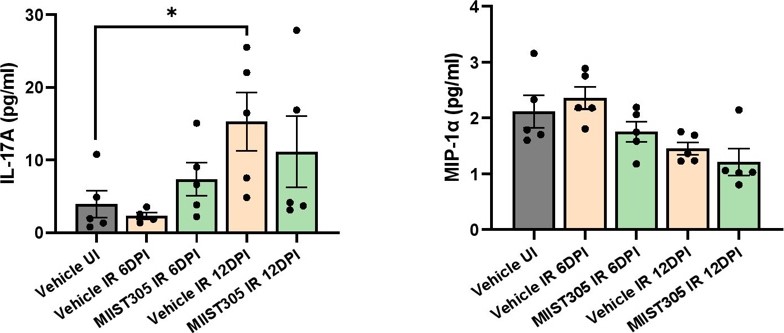


に-27 P28ハL-30

#
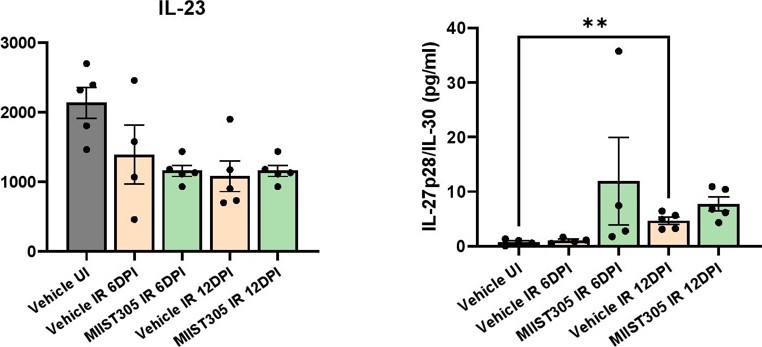
(IW16d)

2 代」

一

S2


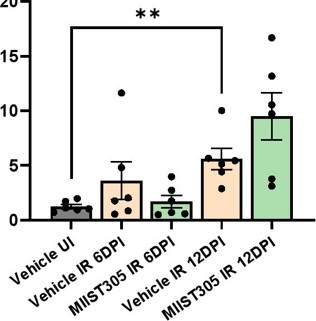


20

IFN-Y

()W

、

5d

)

A-NAI

に-22


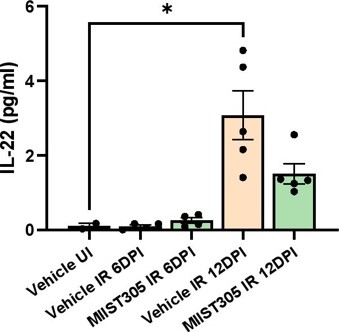


## S3


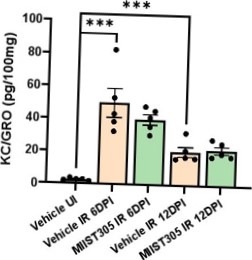
 KC/GRO


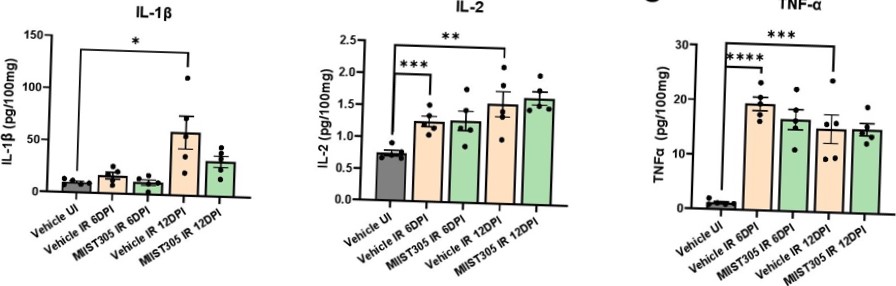


TNF-a

IFN-Y IL-10

IL-17A

| IL-22 | 11--23 | IL-27p28/lL30 |
| --- | --- | --- |

###
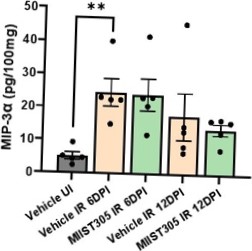

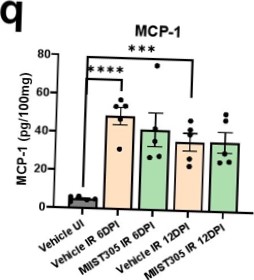

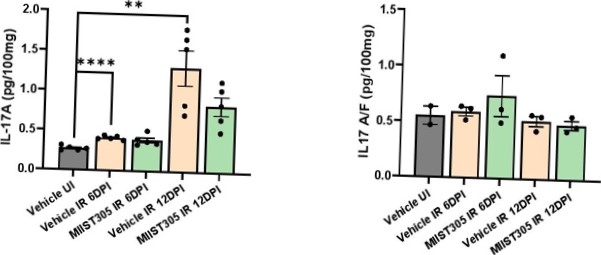

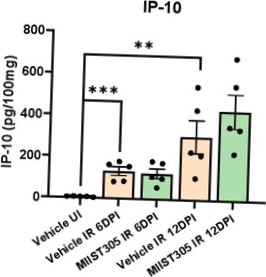

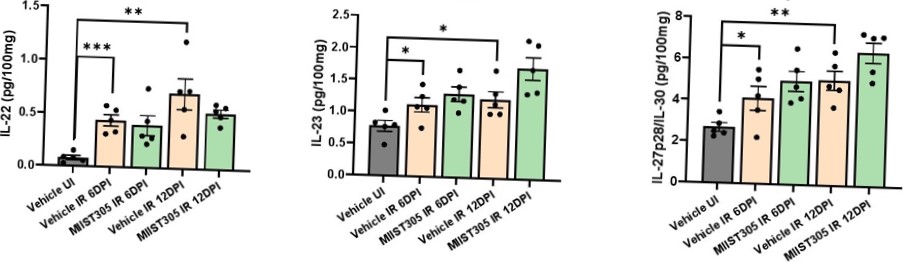

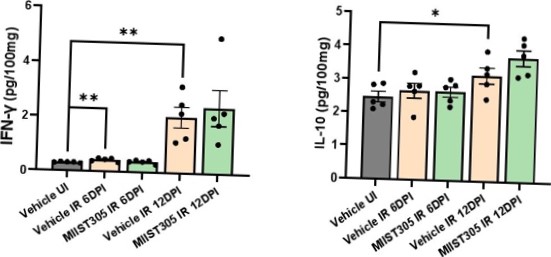
 IL-17A/F

###
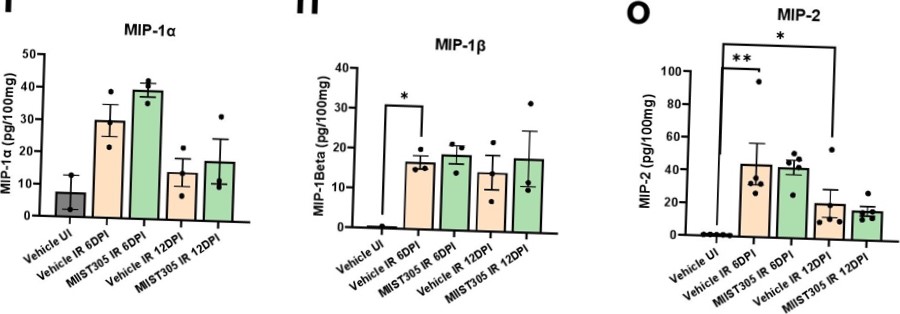
 MIP-3a

Figure

S4


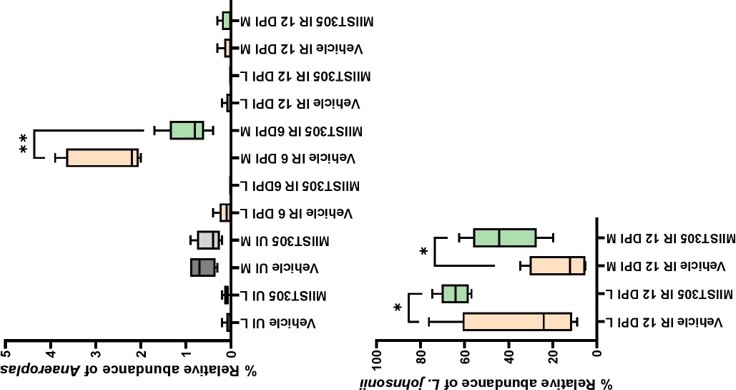


etuse


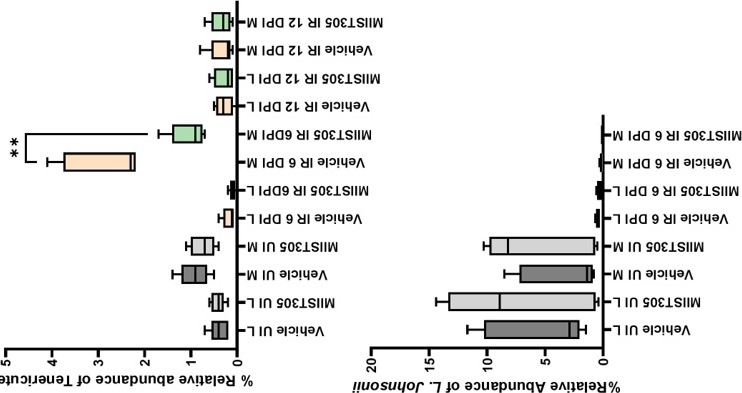


sa
